# Structural imbalance of medical resources amid population mobility and digital empowerment: a study of national and port-developed provinces in China

**DOI:** 10.3389/fpubh.2025.1613293

**Published:** 2025-05-23

**Authors:** Haiwei Fu, Junjie Lu

**Affiliations:** School of Economics and Management, Ningbo University of Technology, Ningbo, China

**Keywords:** medical resources, structural imbalance, population mobility, digital empowerment, China, port-developed provinces

## Abstract

Currently, many cities in China are facing the problem of difficult medical care and expensive medical care, while at the same time a large number of hospitals are on the verge of closing. However, existing literature is unable to adequately explain these phenomena. To fill this gap, this study focuses on the causes of the structural imbalance of medical resources in China from the perspective of population mobility and the governance pathways of digital empowerment. By analyzing the entire country and the port-developed provinces with advanced medical conditions, several key findings are revealed: (1) Different types of medical resources in cities exert differentiated attraction effects on population mobility. High-tier hospitals exert a strong pull on population mobility, while the impact of primary care facilities is not evident; (2) Continuous population movements lead to a structural imbalance of medical resource, characterized by overburdened high-tier hospitals coexisting with underutilized primary care facilities; (3) Economic development has increased people’s emphasis on health and prevention of critical illnesses, making the aforementioned structural imbalance more pronounced in the port-developed provinces of China; (4) Digital empowerment offers a new perspective for addressing these issues. By introducing digital diagnostic devices in primary care facilities, the likelihood of major diseases can be swiftly assessed. This technology not only enables intelligent triage but also effectively mitigates the structural imbalance of medical resources.

## 1 Introduction

Medical resources are essential for urban and regional development. They not only improve people’s physical health but also boost the economy by creating a healthier population (1–3). Studies show that hospitals are at the core of medical resources and can be divided into primary care facilities and high-tier hospitals (4–6). Recently, both types of hospitals in China have seen rapid growth. According to data from the National Bureau of Statistics of China, the total number of healthcare institutions in China was 990,248 in 2015, and by 2024 this number had increased to 1,092,000, which reflects China’s robust expansion in healthcare infrastructure and its emerging prominence on the global stage.

In China, medical resources have been facing serious structural imbalances in recent years. On the one hand, there are a huge number of high-tier hospitals being overcrowded in many cities, making it difficult for patients to get timely appointments (7). On the other hand, primary care facilities often see very few patients, which lead to doctors and medical equipment being underutilized (8, 9). For example, many people have never sought treatment at a primary care facilities throughout their entire lives in some international cities such as Shanghai. To put it more dramatically, there are many children living in cities who cannot even imagine what a primary care facilities looks like (10, 11). As a result, this structure imbalance of medical resource will not only negatively impact the physical well-being of the population, but also hinder the development of the healthcare industry. In order to explore the causes of this phenomenon, researchers have found several factors: First, the rapidly aging population has led to an increase in the number of consultations at high-end hospitals (12, 13). This is mainly because the immunity of the older adult is much worse than that of the young. Second, as the public awareness of health issues rises, more people are seeking high-quality, personalized medical services (14). This shift adds even more pressure on these hospitals (15, 16). Third, cultivating medical professionals takes a considerable amount of time, which means that the supply of qualified professionals often lags behind the expanding market demand (17, 18). In China, the duration of study for an undergraduate medical degree is 5 years, and for a master’s degree graduate student, it reaches 4 years, which is far longer than students of other majors. Fourth, the construction of modern advanced hospitals requires substantial investment, but due to the sluggish real estate market and the continuous decline in government land transfer revenues, persistent fiscal deficits have limited government’s spending on medical infrastructure (19–21).

While previous studies have looked into the reasons behind the shortage of high-tier hospitals in cities from various angles, there are still some important gaps need to be filled. First, many studies overlooked why primary care facilities are underutilized. Second, most scholars neglect that factors such as population aging often lead to a gradual shortage of high-tier hospitals (22–24), while the shortage in China occurred at an alarming rate. This discrepancy suggests that there may be other reasons behind these issues.

With the acceleration of China’s urbanization, population mobility can be one possible driver. According to the 7th National Census of China, the migrant population accounts for 26.6% of the total population. This phenomenon has been a notable part of China’s growth over the last 40 to 50 years since the reforms began in 1978 (25, 26). Early on in China’s economic boom, people primarily moved from inland areas to the coastal areas, attracted by the prospect of higher salaries (27, 28). In the 1980s and early 1990s, the Pearl River Delta region experienced a remarkable influx of people from all over the country. Today, Guangdong Province still leads China in population growth, with cities like Dongguan, Shenzhen, and Zhongshan hosting over half of the area’s permanent residents. However, this pattern have changed notably after the per capita GDP exceeded CNY 70,000 (approximately $9,589, based on an exchange rate of 7.3 CNY/USD). Currently, people’s desire to boost their life expectancy is motivating them to move to cities that provide improved transportation, healthcare, and educational resources (29), which may help explain the sudden and unexpected shortage of high-tier hospitals in China.

In view of this gap, this paper attempts to construct an ERGM model under a systematic analysis framework focused on do people’s preferences for different types of hospitals lead to population mobility and then identifies the impact of population mobility on the structure imbalance of medical resources in China, analyzing the methods for solving the above problem. Compared with the existing literature, this paper made the following contributions. First, it constructed ERGM models to identify the effects of different types of hospitals on population mobility. Second, it illustrated how population mobility has led to a structural imbalance in medical resources over time. Third, it proposed a digital empowerment approach to address these challenges.

## 2 Concept definition and study hypothesis

### 2.1 Medical resources

When discussing medical resources, we generally refer to an interconnected system including hospitals, doctors, technology, and more (30, 31). Narrowing the focus, we can primarily define medical resources as hospitals, which are the most critical component (32). This study mainly refers to medical resources in a narrower view, which specifically means hospitals.

Based on the different populations served, hospitals can be classified into two categories: primary care facilities and high-tier hospitals (33). Specifically, primary care facilities focus on delivering basic medical services for common illnesses, while high-tier hospitals mainly focus on handle more complex illnesses. In most cases, the medical equipment at primary care facilities is more basic (like X-ray machines), and high-tier hospitals mainly apply cutting-edge technology (like CT scanners and MRI machines). In recent years, some high-tier hospitals in major cities in China, like Shanghai, have even started using robotic-assisted surgical devices to enhance the efficiency of surgeries (34, 35). In this study, primary care facilities refer to community health service centers, township hospitals, and first-level hospitals in Chinese cities, while high-tier hospitals include second-and third-level hospitals, specialized hospitals, as well as some international or high-end private hospitals.

### 2.2 Medical resource allocation and population mobility

Whether in developed or developing countries, population mobility is a widespread phenomenon. Unlike natural population growth, population mobility tends to exert sudden shocks on the preexisting structural imbalance of medical resource. Accordingly, this paper adopts a population mobility perspective to investigate the primary causes of structural imbalances of medical resource in Chinese cities.

Recent studies show that population mobility patterns in China have changed significantly (36, 37). Back in the 1980s and early 1990s, as outlined by Lewis’s theory of a dual urban–rural economic structure, most people moved from rural areas to cities, primarily driven by income differences. Many individuals sought better wages and often relocated from inland regions to coastal areas, where thriving exports usually meant higher salaries (38). Since China’s reform and opening up in 1978, coastal regions have gradually developed a labor-intensive, export-oriented industrial structure, thanks in part to their geographical advantages and favorable policies compared to inland areas. However, as economic development continues and living standards improve, the traditional pattern of rural-to-urban and inland-to-coastal population mobility has gradually slowed (39). It has been replaced by a trend in which populations concentrate in large cities offering superior infrastructure, education, and medical resource (40, 41).

As people increasingly prioritize quality of life, different types of hospitals are becoming more distinct in their appeal, which is influencing where people choose to live. This growing focus on quality of life has made higher-tier hospitals quite rare, which in turn makes them more valuable (42). In Chinese port-developed provinces, many city has only a few top-tier hospitals, which makes them more attractive to people. Thus, this study proposed the following hypotheses:

*H1a:* More primary care facilities in a city will not really boost its appeal for attracting people.

*H1b:* Conversely, more high-tier hospitals are likely to make a city much more appealing to potential newcomers.

### 2.3 Structural imbalance of medical resources

From our earlier discussion, in some cities, the need for high-tier hospitals often grows much faster than what you’d expect from just the natural increase in population. However, training a medical professional takes a long time, and building hospital facilities often requires a lot of money, which makes it tough to quickly scale up. When demand spikes but supply cannot keep up, we would facing serious shortages of high-tier hospitals. In China, if you want to register at a high-end hospital, you typically need to wait several hours at least. Moreover, with more and more people using apps to make appointments, there is an increasing phenomenon of “traffic jam” in high-tier hospitals (43, 44). Meanwhile, primary care facilities are still not being used to their full potential in various cities throughout China. And as more people flock to areas with plentiful high-quality medical resources, this structure imbalance will only worsen, thus creating a self-perpetuating cycle. This is why, when we pass by a primary care facility on the street in Chinese cities, we might notice that there are hardly any patients inside, and doctors and nurses might even be dozing off at desks or playing on their phones.

This issue is not only widespread across the country but is especially noticeable in economically developed regions. Much of this is tied to the rapid growth of China’s economy, which has significantly improved overall living standards and raised public awareness about health issues. Research has shown a strong positive relationship between levels of economic development and health concerns (45, 46). According to the latest data from the National Bureau of Statistics of China, the per capita GDP in 2024 reached 95,700 RMB, approximately 13,700 USD (based on an assumed exchange rate of 1:7). In the port-developed provinces along the coast, the level of economic development is leading, and residents’ attention to health issues is expected to be even more pronounced. Zhejiang Province, recognized as the most economically advanced port province, reported a per capita GDP of 135,600 RMB in 2024, far exceeding the national average. Meanwhile, Zhejiang Province has become a pilot province for “common prosperity” due to its port advantages. Therefore, the phenomenon of structural imbalance of medical resources will be more pronounced in these port-developed provinces.

Given this reasoning, this study proposed the following hypotheses:

*H2a:* The population mobility driven by high-tier hospitals will result in a structural imbalance of medical resources in China.

*H2b:* The structural imbalance mentioned above will be more evident in port-developed provinces.

### 2.4 Digital empowerment

Although most people choose to receive treatments at high-tier hospitals, the majority of patients visiting these facilities in China are diagnosed with routine illnesses (47), which creates a paradox: even though primary care facilities can effectively treat routine illnesses, patients are willing to spend excess time and money at high-tier hospitals. To explain the above phenomenon, some studies have suggested that people are often willing to take measures to reduce their risk of critical illnesses when faced with health concerns (48–50).

Therefore, considering the above viewpoints, this study held that primary care facilities need digital tools using machine learning to diagnose diseases more accurately. By this method, those with routine issues will be able to receive care at local health service centers while significant disease risk cases can be referred to higher-tier hospitals (51, 52). Thus, we proposed the following research hypothesis:

*H3:* Digital empowerment of primary care facilities can help balance out the different kinds of medical resources in China.

**Figure 1:**
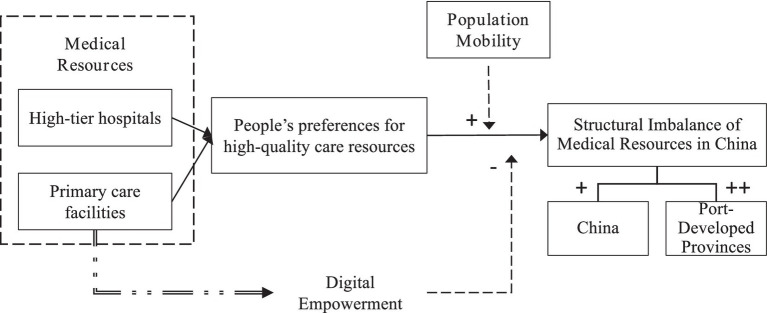
Research framework.

To sum up, the complete research framework is shown in Figure 1. In this framework, “+” represents a promoting effect, “++” represents an even stronger promoting effect, and “−” represents an inhibitory effect.

## 3 Research methodology

### 3.1 Data collection

In this study, we examine how the structural imbalance of medical resources in China occurs from the perspective of population mobility. The data on population mobility comes from two sources. The data for 2023 comes from the population mobility data provided by Amap, and the data for 2017 was collected from the large-scale national migration sample survey (CMDS) carried out by the National Health Commission of the People’s Republic of China. To further obtain the data on medical resources, we download a large amount of data from the websites of the Health Committees of each sample cities in China. At the same time, we also search a great amount of data from the China Health Statistics Yearbook. Recognizing that population mobility will be influenced by other various factors in reality, we further collect data from the China Urban Statistical Yearbook and the Zhejiang Statistical Yearbook.

Specifically, the population mobility data from Gaode Map in 2023 mainly reflects the population movement between prefecture-level cities, which covers the movement of people between 288 prefecture-level cities across China. It should be noted that in China, a prefecture-level city (like Hangzhou City in Zhejiang Province) is a type of city whose scale lies between provinces (like Zhejiang Province) and county-level cities (like Chunan County in Hangzhou city, Zhejiang Province).

In order to figure out whether the structural imbalance of medical resources caused by population mobility is more severe in port-developed provinces with relatively higher levels of economic development and health awareness (the hypothesis H2b), we choose Zhejiang Province as our another research sample. This is because Zhejiang province is renowned for its developed port trade and is one of the most economically developed provinces in China. In this part, population mobility primarily occurs between the 88 county-level cities in Zhejiang Province. It is important to note that in China, a province consists of multiple prefecture-level cities, and each prefecture-level city consists of several county-level cities. Therefore, county-level cities can be regarded as the smallest city units.

**Figure 2:**
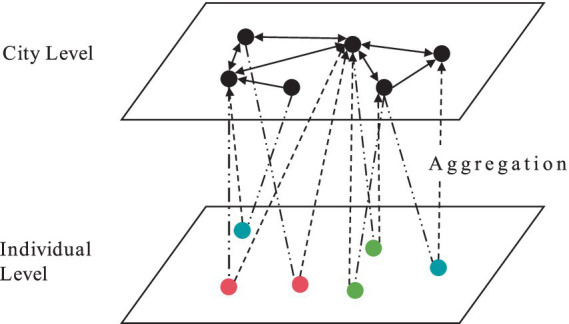
Data transformation process.

Figure 2 demonstrates the transformation process from micro-level data at the individual level in CMDS survey to macro-level data at the city level. In this study, all population mobility data from the CMDS survey were organized into an edge list format, arranged according to the movement of individual i from city x to city y. As shown in Figure 2, the data at the city level is an aggregation of the individual data. For example, the population mobility from city A to city B is measured by counting how many individuals in the edge list moved from city A to city B.

### 3.2 Method selection

The Exponential Random Graph Model (ERGM) is the core analytical method of this study, which is used to examine hypotheses H1 and H2. In academia, some scholars have used this model to analyze interpersonal relationship networks, such as research collaboration among the scholars (53). Besides, some researchers have also used this method to analyze trade networks between countries across the World (54–56). The reason the ERGM model is widely used in network analysis is that it offers a robust way to analyze how multiple factors shape network formation (57, 58). It generally describes the probability distribution of the network based on p influencing factors, where the normalizing constant “k(*θ*)” makes sure that the probability mass function “p_θ_(x)” sums to 1 across the entire network.


$$\mathrm{Pr}(X=x|\theta )=p_{\theta }(x)=\frac{1}{k(\theta )}\exp\{\theta_{1}z_{1}(x)+\theta_{2}z_{2}(x)+\cdots +\theta_{p}z_{p}(x)\}$$

The ERGM model constructed in this study is formulated as follows. In this formulation, z₁(x), z₂(x), and z₃(x) represent the covariates for the network’s endogenous structure, individual member attributes, and exogenous networks, respectively (59, 60). First, endogenous structural variables are mainly used to test the regular structural patterns that emerge during network formation. Second, individual attribute variables measure how node characteristics shape network formation and are divided into sender and receiver effects. The former explores whether a stronger attribute makes a node more inclined to form connections with others, while the latter evaluates whether a salient attribute increases the likelihood that a node will be connected by others. Third, exogenous network variables are aimed to assess whether other external networks influence the formation of the focal network.

Therefore, θ₁, θ₂, and θ₃ are used to measure which kind of variable will significantly influence the network formation (Table 1). In our study, the number of primary care facilities and the number of top-tier hospitals in a city are individual attribute variables. When the coefficient of the variable in Table 1 is statistically significant, the factor corresponding to which would thus be conducive to attracting population to the city.

**Table 1:** Main parameters of the ERGM and their meanings.

Parameter	Structure	Explanation
Endogenous structural variables		
Edges	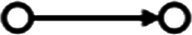	Edges is used to assess whether the network formation exhibits randomness.
Individual attribute variables		
Receiver effect	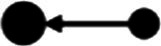	Receiver effect examines the attractiveness of city characteristics for attracting population inflow.
Exogenous network effect		
Network covariates	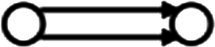	The exogenous network effect examines whether cities with stronger other network relationships are more likely to promote population migration.

Besides, to validate hypotheses H2 and H3, this study further apply network centrality analysis by using a mix of tools, including UCINET, ArcGIS, and R.

### 3.3 Variable measurement

In our ERGM model, the dependent variable is the population mobility network, while the independent variables are categorized into three types (Table 2).

**Table 2:** Description of variables in the ERGM model.

Variable	Description
Endogenous structural variables	
Edges	Used to assess whether the network is generated randomly.
Individual attribute variables	
Nodeicov.PCF	Impact of primary care facilities on city attractiveness (Unit: Count).
Nodeicov.HTH	Impact of high-tier hospitals on city attractiveness (Unit: Count).
Nodeicov.GDP	Economic development level measured by Gross Domestic Product (Unit: 100 Million RMB).
Nodeicov.INT	Internet development assessed through telecommunications revenue (Unit: 100 Million RMB).
Nodeicov.ENV	Environmental quality quantified by Annual Mean Concentration of PM2.5 (Unit: μg/m^3^).
Nodeicov.UR	Urbanization rate reflecting rural-to-urban migration (Unit: %).
Nodeicov.EDU	Number of high school students (Unit: 10,000 students).
Exogenous network effect	
Edgecov.GeoProx	Geographic proximity effect based on the inverse of Euclidean distance (Unit: Euclidean distance’s inverse).
Edgecov.CulProx	Cultural proximity based on shared dialects between cities (Unit: 0 or 1).


$$\mathrm{Pr}(X=x|\theta )=p_{\theta }(x)=\frac{1}{k(\theta )}\exp\{\theta_{1}z_{1}(x)+\theta_{2}z_{2}(x)+\theta_{3}z_{3}(x)\}$$

This study selected Edges as its indicator. Specifically, if the coefficient of Edges is significantly negative, the formation of the population mobility network can be considered non-random.

In terms of Individual Attribute Variables, we chose the number of primary care facilities (Nodeicov.PCF) and high-tier hospitals (Nodeicov.HTH) in cities to evaluate their reception effects. At the same time, population mobility can be affected by a range of factors, including economic development level (Nodeicov.GDP) (61), urbanization rate (Nodeicov.UR), environmental quality (Nodeicov.ENV), internet infrastructure development (Nodeicov.INT), and education level (Nodeicov.EDU). The reason for selecting the above control variables is that studies have shown that factors such as environmental quality, internet connectivity, and educational resources will significantly affect population mobility as the living standards improve (62, 63).

In terms of network covariates, we selected geographic proximity effects (Edgecov.GeoProx) and cultural proximity effects (Edgecov.CulProx) as control variables. First, geographic proximity is measured by the inverse of the Euclidean distance between cities. Second, cultural proximity is assessed based on whether the dialects spoken between cities are the same. If two cities share the same dialect, they are marked as having a strong cultural connection. In contrast, if they reside in different dialect regions, they are marked as having a weaker cultural relationship. Typically, people tend to move between areas that share cultural similarities.

## 4 Data analysis and results

### 4.1 ERGM analysis at the national level

In the ERGM analysis at the national level, three different models were constructed in Table 3. The results from the analysis indicated that Model 3 had the lowest scores for both the Akaike Information Criterion (AIC) and Bayesian Information Criterion (BIC), which suggests that Model 3 demonstrates the highest goodness of fit.

**Table 3:** ERGM estimation results at the national level.

Variables	Model 1	Model 2	Model 3
Edges	−5.1234^***^ (0.0501)	−5.1204^***^ (0.0501)	−5.0613^***^ (0.0503)
Nodeicov.PCF	0.0001 (0.0001)	0.0001 (0.0001)	0.0001 (0.0001)
Nodeicov.HTH	0.0158^***^ (0.0006)	0.0158^***^ (0.0006)	0.0122^***^ (0.0006)
Nodeicov.GDP	0.0000^***^ (0.0000)	0.0000^***^ (0.0000)	0.0000^***^ (0.0000)
Nodeicov.INT	0.0076^***^ (0.0006)	0.0076^***^ (0.0006)	0.0049^***^ (0.0005)
Nodeicov.ENV	−0.0076^***^ (0.0013)	−0.0075^***^ (0.0013)	−0.0132^***^ (0.0013)
Nodeicov.UR	–	−0.0001^**^ (0.0000)	−0.0001^**^ (0.0000)
Nodeicov.EDU	–	–	0.0503^***^ (0.0026)
Edgecov.GeoProx	5898.2958^***^ (49.7692)	5900.3391^***^ (49.7790)	5826.3604^***^ (50.2868)
Edgecov.CulProx	–	–	0.6122^***^ (0.0807)
AIC	56941.7975	56935.1627	56479.7459
BIC	57007.0546	57009.7422	56572.9703
N (sample size)	288	288	288

The table displays the regression coefficients along with their standard errors. The significance levels are noted as: *** for *p* < 0.001, ** for *p* < 0.01, and * for *p* < 0.05.

In Model 3, the coefficient of primary care facilities is not significant (Nodeicov.PCF = 0.0001, *p* > 0.05). The result has validated Hypothesis H1a, which suggests that cities with a greater number of primary care facilities do not exhibit a noticeable attraction for population mobility. In contrast, the coefficient of high-tier hospitals is significantly positive (Nodeicov.HTH = 0.0122, *p* < 0.001), indicating that cities with a larger number of high-tier hospitals have a notable appeal for population inflow, thus supporting hypothesis H1b. High-tier hospitals can effectively cater to the people’s demand for increasingly higher quality of life. Because of this, they play a vital role in drawing people to a city.

The result of the control variables are consistent with the findings from previous studies. First, the coefficient of Edges is significantly negative (Edges = −5.0613, *p* < 0.001), indicating that the formation of the population mobility network is not random. Second, the coefficient of Nodeicov.GDP is significantly positive (Nodeicov.GDP = 0.0000, *p* < 0.001), indicating that economically developed cities tend to attract more people compared to underdeveloped areas. Third, the coefficient of Nodeicov.UR is significantly negative (Nodeicov.UR = −0.0001, *p* < 0.01), indicating that cities with higher urbanization rates have a lower attraction for population inflow. This may be because these cities often have higher population densities, which will lead to an increased level of congestion. Fourth, the coefficient of Nodeicov.ENV is also significantly negative (Nodeicov.ENV = −0.0132, *p* < 0.001), which implies that cities suffering from poor air quality will see a notable decline in their attractiveness for new residents. This is because the incidence and mortality rates of lung cancer are the highest among all cancers in China, and air quality is an important factor influencing the incidence of lung cancer. Besides, both of Nodeicov.INT and Nodeicov.EDU have significantly positive coefficients (Nodeicov.INT = 0.0049, *p* < 0.001; Nodeicov.EDU = 0.0503, *p* < 0.001), which suggests that cities with rich internet infrastructure and educational resources are much more appealing to populations.

As population mobility continues, the demand for high-tier medical resources is rising rapidly, far exceeding the growth rate of demand brought about by the natural population growth rate of within 3% per year. This is mainly because population mobility between cities in China is unrestricted, meaning that a city could see an influx of over a hundred thousand or even a million people in a single year. However, because the supply of high-tier hospitals is rigid, it’s not feasible to quickly build a large number of them within a short period. As a result, it will become difficult to respond quickly to the surge in demand, which leads to a supply shortage of high-tier hospitals. Meanwhile, cities with a large amount of primary care facilities fail to attract population mobility. Even if the population increases for some reason, people will seek high-tier medical resources and are unlikely to visit primary care facilities, which would ultimately lead to a surplus in the supply of primary care facilities.

**Figure 3:**
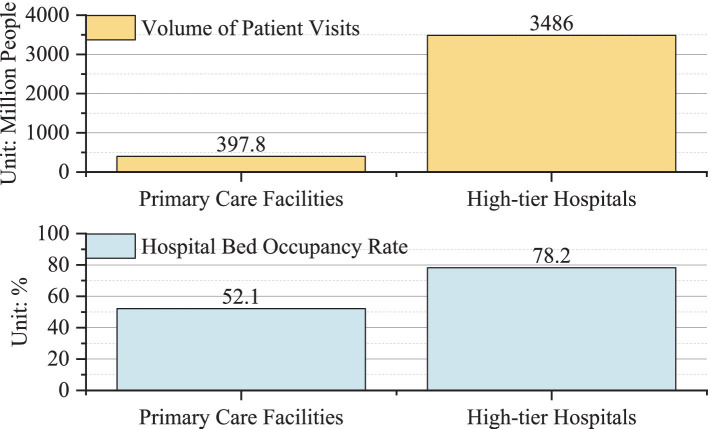
Structural imbalance of medical resources in Chinese cities in 2021.

Figure 3 clearly illustrates the structural imbalance of medical resources mentioned above. In 2021, there were a total of 34.86 million patients who had received diagnosis and treatment in high-tier hospitals in China, while only 3.978 million patients received diagnosis and treatment in primary care facilities. Interestingly, the bed occupancy rate in high-tier hospitals sits at 78.2%, while primary care facilities lag behind at just 52.1%. In Chinese cities, many primary care hospitals have opted to close temporarily during the day due to a lack of patients, which leaves many healthcare workers with no choice but to seek other ways to earn a living, such as doing delivery jobs or providing domestic help. Consequently, hypothesis H2a has been validated.

### 4.2 ERGM analysis at the provincial level

In this part, we chose Zhejiang Province as our research sample. The main reason is that Zhejiang Province is one of the regions in China that has achieved economic prosperity and even become a first pilot area for common prosperity across China due to the development of its ports and international trade. This case primarily aims to test hypothesis H2b, which examine whether the structural imbalance of medical resources is more apparent in port-developed provinces.

The analysis at the provincial level includes three models, but it should be noted that due to the less comprehensive statistical data at the provincial level compared to the national level, the number of control variables in Table 4 is lower than that in Table 3. Model 1 includes most of the variables other than geographic proximity (Edgecov.GeoProx) and cultural proximity (Edgecov.CulProx). Model 2 introduces geographical proximity and Model 3 further adds cultural proximity as an control variable. The empirical results show that Model 3 achieves the lowest AIC and BIC values, which indicates that it provides the best fit for the data. According to the results of Model 3, the coefficient of primary care facilities remains insignificant (Nodeicov.PCF = 0.0004, *p* > 0.05), while the coefficient of high-tier hospitals remains significantly positive (Nodeicov.HTH = 0.0513, *p* < 0.001), consistent with the estimates presented in Table 3. This further validates hypotheses H1a and H1b, which state that different types of medical resources exert differentiated attraction effects on population mobility.

**Table 4:** ERGM estimation results at the provincial level.

Variables	Model 1	Model 2	Model 3
Edges	−3.5704^***^ (0.0952)	−3.7224^***^ (0.0984)	−3.6741^***^ (0.0997)
Nodeicov.PCF	0.0006 (0.0004)	0.0006 (0.0004)	0.0004 (0.0004)
Nodeicov.HTH	0.0502^***^ (0.0093)	0.0520^***^ (0.0094)	0.0513^***^ (0.0094)
Nodeicov.GDP	0.0000 (0.0001)	0.0000 (0.0001)	0.0001 (0.0001)
Nodeicov.INT	0.0841^***^ (0.0089)	0.0833^***^ (0.0091)	0.0837^***^ (0.0091)
Edgecov.GeoProx	–	1.1873^***^ (0.1123)	1.3447^***^ (0.1305)
Edgecov.CulProx	–	–	−0.2533^*^ (0.1048)
AIC	4403.4285	4308.3034	4304.2947
BIC	4438.1447	4349.9629	4352.8974
N (sample size)	88	88	88

The table displays the regression coefficients along with their standard errors. The significance levels are noted as: *** for *p* < 0.001, ** for *p* < 0.01, and * for *p* < 0.05.

The signs of the other control variables align with those in Table 3. Notably, the coefficient of Nodeicov.GDP has lost its significance (Nodeicov.GDP = 0.0001, *p* > 0.05), which may be due to the balanced economic development across cities in Zhejiang Province. Generally speaking, population mobility tends to occur between regions with significant economic disparities, rather than between areas that are relatively similar. In other words, just as sufficient force is required to overcome static friction and initiate the motion of an object, two cities must exhibit significant economic differences to drive population mobility. Besides, the coefficient of Edgecov.CulProx is significantly negative (Edgecov.CulProx = −0.2533, *p* < 0.05). This may be due to the higher education level and the widespread use of Mandarin in Zhejiang Province.

### 4.3 Mechanism analysis in port-developed provinces

**Figure 4:**
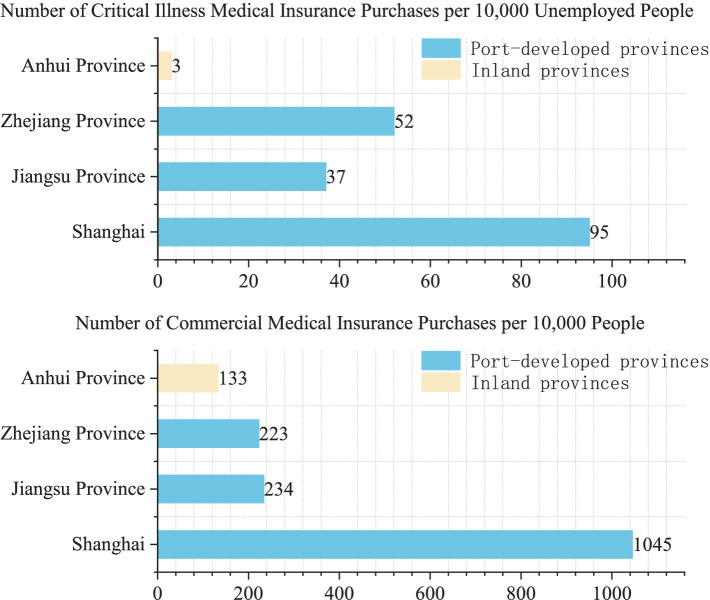
Medical insurance purchasing among residents in the Yangtze River Delta in China.

Further analysis is shown in Figure 4. In the Yangtze River Delta urban agglomeration in China, there are three provinces and one province-level municipality, namely Shanghai, Jiangsu, Zhejiang, and Anhui. Among these areas, Shanghai, Jiangsu, and Zhejiang are coastal regions with ports and relatively developed economies, while Anhui is an inland region without a port and exhibits slower economic development. It is clear that people in port-developed areas pay much more attention to health compared to those in inland areas. For example, in terms of individual commercial medical insurance purchases, the level in port-developed areas exceeds 200 individuals per 10,000 people. However, the rate is only 133 per 10,000 in Anhui Province. Similarly, the number of critical illness medical insurance purchases per 10,000 unemployed people is far higher in port regions than in the inland areas. In short, economic development has made people pay more attention to disease prevention, which will results in a more pronounced structural imbalance of medical resources in port-developed provinces.

**Figure 5:**
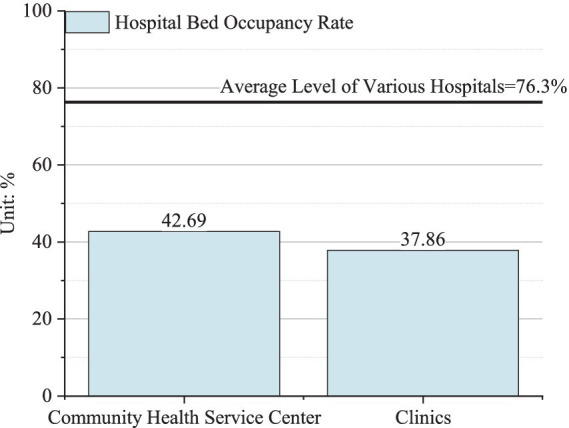
Structural imbalance of medical resources in Zhejiang Province in 2021.

As shown in Figure 5, the bed occupancy rates of community health service centers and clinics in 2021 in China were 42.69 and 37.86%, which are far below the provincial average of 76.3% and significantly lower than the 52.1% occupancy rate of primary care facilities shown in Figure 3. This indicates that the structural imbalance of medical resources is even more pronounced in the economically and port-developed Zhejiang Province compared to other regions across the country. Therefore, hypothesis H2b is validated, which indicates that economic development has intensified people’s focus on health and their preference for high-tier hospitals and further amplified the structural imbalance in the port-developed provinces of China.

To illustrate the mechanisms mentioned above dynamically, this study further conducted the following analysis.

**Figure 6:**
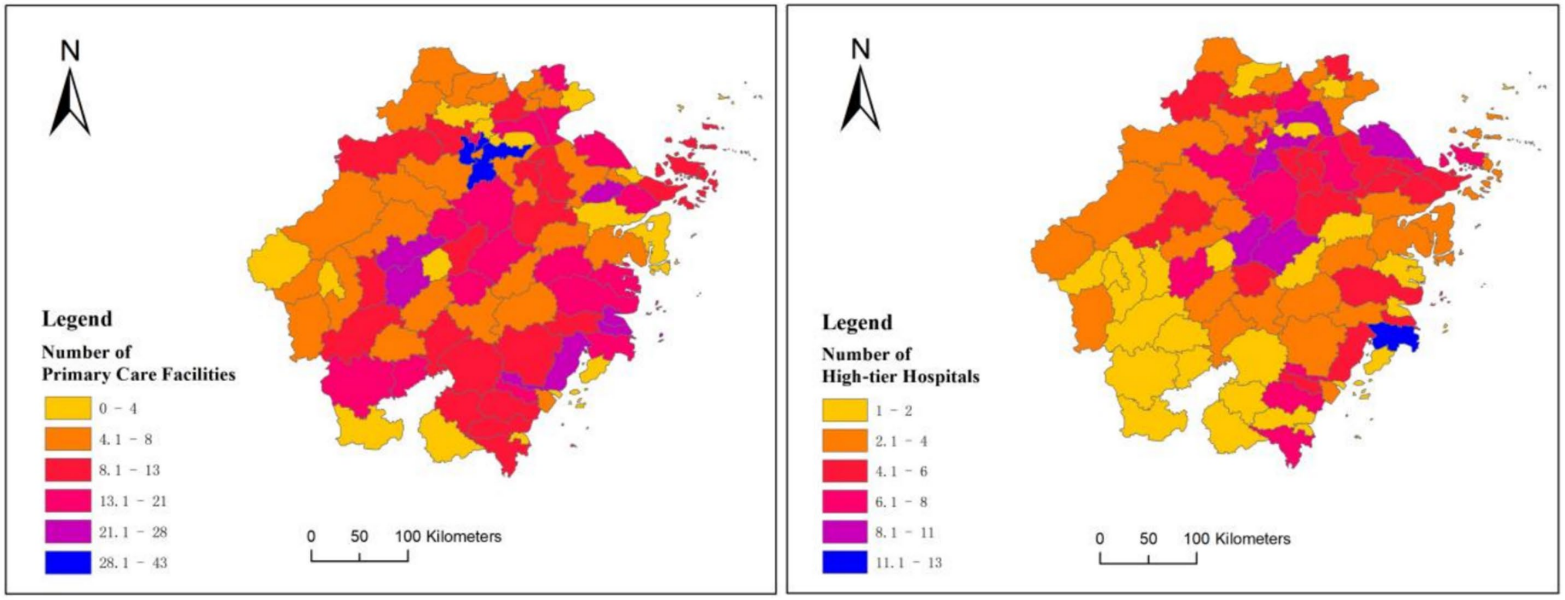
Distribution of primary care facilities and high-tier hospitals in cities of Zhejiang Province.

As shown in Figure 6, the left side shows the amount of primary care facilities of each county-level city in Zhejiang Province, while the right side shows the amount of high-tier hospitals. It’s clear that the southwestern part of Zhejiang has a higher concentration of primary care facilities, as shown by the overall reddish tone on the map, which stands out in comparison to the predominantly orange areas in the north. However, when we look at the right chart, it reveals that the southwestern region has significantly fewer high-tier hospitals, represented by a yellow hue on the map, which is dramatically less than the red areas in the northern part of the province.

**Figure 7:**
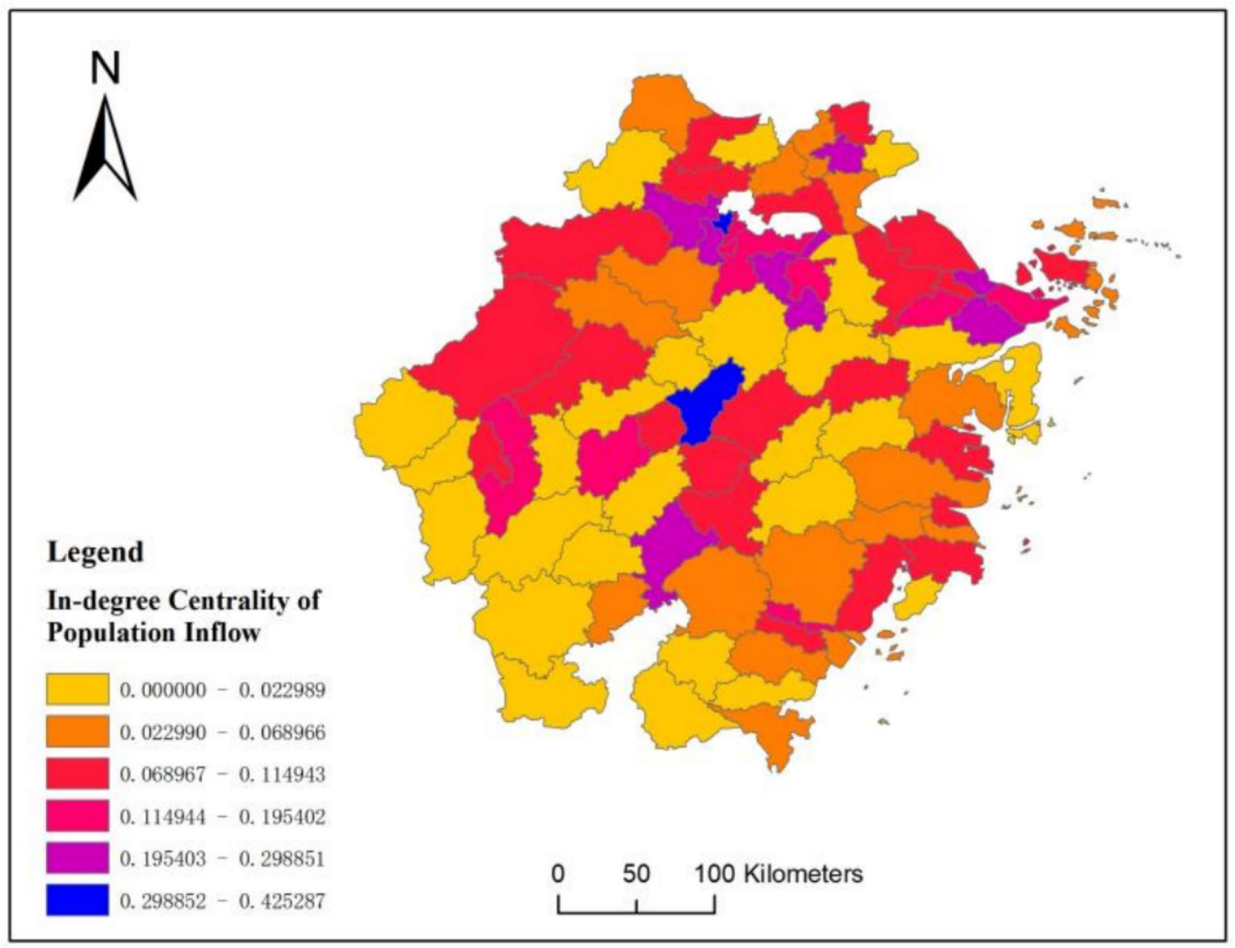
In-degree centrality of population mobility in cities of Zhejiang Province.

Figure 7 further displays the standardized in-degree centrality of population movement across various cities in Zhejiang Province. It’s apparent that the northern region shows higher standardized in-degree centrality compared to the southwestern area. For example, it has reached above 0.068 in regions such as Ningbo and Hangzhou. More importantly, the color distribution pattern we have seen in Figure 7 aligns well with the color distribution pattern seen on the right side of Figure 6, which further validate hypotheses H1 and H2.

At the same time, the population mobility shown in Figure 7 will further exacerbate the structural imbalance of medical resources in Zhejiang Province. As more people move into the northern part of Zhejiang Province, the southwestern region is experiencing population outflow, exacerbating the structural imbalances in medical resources across the province. In some county-level cities in Zhejiang Province, high-tier hospitals have captured almost 80% of patients, leaving only a handful for primary care facilities.

### 4.4 The role of digital empowerment

**Figure 8:**
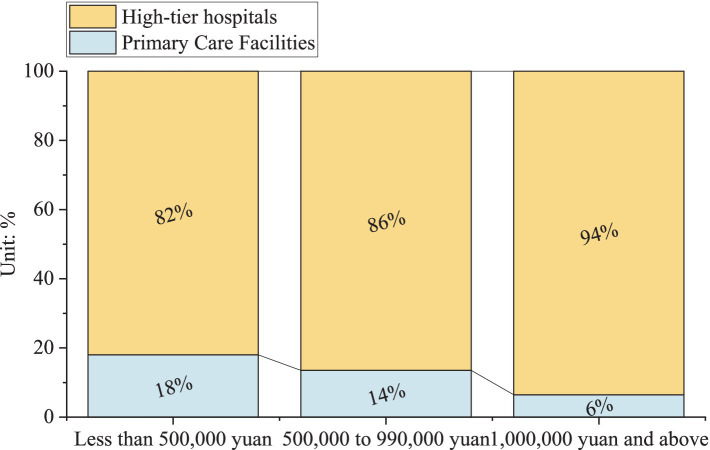
Distribution of medical equipment valued over 10,000 yuan in primary care facilities and high-tier hospitals in China in 2022.

In this section, Table 5 showcases data conducted by researchers at Zhongnan University of Economics and Law from 2016 to 2022 (64). Notably, about 76.4% among the respondents believe that the quality of medical equipment is lacking. Figure 8 further illustrates the distribution of medical equipment valued over 10,000 yuan in primary care facilities and high-tier hospitals in China in 2022. It is clear that high-tier hospitals possess significantly more medical devices valued at this amount compared to primary care facilities. At the same time, as the value of the equipment increases, we can see a noticeable decline in the proportion of devices found in primary care facilities. Specifically, among equipment valued at less than 500,000 yuan, the share of equipment owned by primary care facilities accounts for 18% of the total. This share decreases to 14% for equipment valued between 500,000 and 990,000 yuan, and further drops to just 6% for equipment over 1 million yuan. As a result, primary care facilities face a significant service capacity gap. Consequently, it is no surprise that many people hesitate to seek diagnoses and treatments there.

**Table 5:** Survey of weaknesses in primary care facilities.

Issues identified (%)			
Unsatisfactory conditions of the equipment	Shortage of medications	Inadequate medical skills	Poor attitude of doctors
76.4	69.6	69.4	20.9

Therefore, digital empowerment may be at work. This is because digital devices employed in machine learning-based risk probability models can meet patients’ needs for managing health risks (65–67). As primary care facilities can provide diagnostic services comparable to those of high-tier hospitals, this will ultimately help to correct the imbalance in the structure of medical resources in China.

For instance, at the Fengxian Community Health Service Center in Shanghai, an AI-assisted imaging ultrasound device was introduced in 2025. Using this machine for examinations is like having an doctor nearby constantly giving audio and visual alerts when abnormalities are detected. Since this device was introduced, over 1,000 patients have undergone ultrasound screenings for breast cancer as of April. Through this approach, this center is able to generate substantial income and makes healthcare more convenient for local residents.

Another example is the Pozi Street Community Health Service Center in Changsha. Powered by DeepSeek’s large-model computing and deep-learning capabilities, the system performs real-time data analysis to track patients’ health metrics, promptly flag abnormal readings early warnings about potential major disease. So far, the center has handled a total of 29,048 face-to-face consultations, which has raised patient satisfaction to 97% and greatly enhanced the appeal of primary care facilities. Thus, hypothesis H3 is validated.

## 5 Conclusions and policy implications

In China and its port-developed provinces, a large number of high-tier hospitals are experiencing severe shortages, while primary care facilities remain underutilized. To explore its underlying causes, this paper presented a research framework to examine how population mobility contributes to this phenomenon and propose strategies to address the issue.

Research have shown that high-tier hospitals play a vital role in meeting people’s needs for health investments and risk management, thereby can help a region attract a large inflow of population. In comparison, the role of primary care facilities is not as remarkable. The ERGM models conducted on 288 prefecture-level cities in China and 88 county-level cities in Zhejiang Province have both confirmed this mechanism. More crucially, unlike natural population growth, population mobility will create a sudden and explosive impact on the existing supply and demand market for medical resources. Consequently, high-tier hospitals will face severe supply shortages, while primary care facilities often experience apparent supply surpluses. In addition, since higher economic growth often signifies greater public health awareness, this structural imbalance of medical resources is often more pronounced in port-developed provinces in China (68, 69). This is because port areas often have well-established medical and transportation facilities (7, 70–72). Therefore, people are more inclined to seek the best medical conditions, which exacerbates the differences in the attraction of various types of medical resources to the population.

Further empirical analysis have indicated that the aforementioned phenomenon mainly stems from primary-level hospitals’ inability to provide advanced diagnostic and treatment equipment. As a result, most individuals end up seeking treatment for even minor health issues (like colds) at more advanced hospitals (73, 74). To tackle this problem, digital empowerment could be the key. Through AI-assisted diagnosis and remote expert consultations, primary care hospitals can compensate for their technological shortcomings and rebuild patient trust. This, in turn, will enable patients with common ailments to receive treatment on-site while reducing the long waiting times and costs typically associated with high-tier hospitals. In addition, for more serious condition, the patient can be quickly referred to higher-tier hospitals for specialized care.

In China, the closure of numerous primary care facilities has forced society to reevaluate the logic of medical resource distribution, prompting people to question: Is primary healthcare doomed to become a victim? It should be noted that the essence of any hospital is to safeguard lives, not to engage in cold market competition (75, 76). After all, the older adult and chronic disease patients who remain in remote areas also need accessible medical services. Nowadays, many developing countries around the world are also confronting these issues, especially in the context of accelerating population aging (77–79). Therefore, it is crucial to harness the latest advancements in artificial intelligence and introduce digital tools in primary care facilities, which can enhance patient triage and improve the overall efficiency of healthcare services.

## Data Availability

The data analyzed in this study is subject to the following licenses/restrictions: the raw data supporting the conclusions of this article will be made available by the authors, without undue reservation. Requests to access these datasets should be directed to ljj_2018autumn@163.com.
